# High-affinity promotor binding of YhaJ mediates a low signal leakage for effective DNT detection

**DOI:** 10.3389/fmicb.2024.1510655

**Published:** 2025-01-03

**Authors:** Myeongbin Kim, Ryun Kang, Hye Min Park, Eun Bi Cho, Hye Rim Lee, Seong Eon Ryu

**Affiliations:** Department of Bioengineering, College of Engineering, Hanyang University, Seoul, Republic of Korea

**Keywords:** YhaJ-DBD, DNT detection, low-signal-leakage, crystal structure, biosensor

## Abstract

The YhaJ transcription factor responds to dinitrophenol (DNT) and its metabolic products. The YhaJ-involving cells have been exploited for whole-cell biosensors of soil-buried landmines. Such biosensors would decrease the damage to personnel who approach landmine fields. By the structure determination of the DNA-binding domain (DBD) of YhaJ and the structure-guided mutagenesis, we found that the mutation increasing the DNA binding affinity decreases the signal leakage in the absence of an effector, resulting in a significant enhancement of the response ratio for the DNT metabolite detection. The decrease in signal leakage explains the LysR-type transcriptional regulators’ (LTTRs’) unique mechanism of signal absence repression by choosing between two different activation binding sites. We showed that the biosensor performance enhancement by the decrease in signal leakage could combine with the previous signal-enhancing mutations. The novel mechanism of performance enhancement of YhaJ shed light on bacterial transcription regulation and the optimization of biosensors that involve the large family of LTTRs.

## 1 Introduction

LysR-type transcriptional regulators (LTTRs), which constitute one of the largest protein families in prokaryotes, regulate transcription by sensing specific effector molecules (Maddocks and Oyston, 2008; Giannopoulou et al., 2021; Alhadrami, 2018; Baugh et al., 2023). Two conserved domains in LTTRs, the N-terminal DNA binding domain (DBD) and the C-terminal effector binding domain (EBD) are connected by a single linker helix (LH). The effectors for LTTRs include benzoate, p-toluenesulfate, salicylate, reactive oxygen species, and citrate (Ezezika et al., 2007; Monferrer et al., 2010; Devesse et al., 2011; Choi et al., 2001; Chen et al., 2018). The binding of the specific effector molecule to EBD leads to a conformational change in the effector binding domain leading to the transition of the LTTR homotetramer from the closed form to the open form (Maddocks and Oyston, 2008; Choi et al., 2001; Craven et al., 2009; Jo et al., 2019; Monferrer et al., 2010). The tetrameric transition causes a change in the DNA binding involving both the recognition binding site (RBS) and the activation binding site (ABS) (Maddocks and Oyston, 2008; Baugh et al., 2023; Alanazi et al., 2013). The effector binding-mediated changes in LTTRs bend the target DNA for transcription regulation (Giannopoulou et al., 2021; Baugh et al., 2023).

Because of their sensitivity to specific effector molecules, LTTRs have been studied as a component for whole-cell biosensors (Della Corte et al., 2020; Smirnova et al., 2004; Yagur-Kroll et al., 2014). Cells transformed with plasmids containing promoters regulated by LTTRs were linked to reporter genes, including a fluorescent protein or a luciferase, emitting signals depending on effector molecules. YhaJ, which is an LTTR family member, has been exploited for the detection of explosives such as soil-buried landmines (Shemer et al., 2018; Shemer et al., 2017; Shemer et al., 2020; Shpigel et al., 2021). YhaJ is involved in pathogenic *E. coli* virulence by regulating the degradation of quinol-like compounds (O’Boyle et al., 2023; Palevsky et al., 2016). Small amounts of dinitrophenol (DNT) byproduct vapor are emitted from the landmines and can be detected by YhaJ-based biosensors. However, to be used as an efficient biosensor, the wild-type YhaJ needs improvements due to its low sensitivity to effectors. Several studies reported an enhanced response by modifying the YhaJ-regulated y*qjF* promotor or the YhaJ protein itself (Shemer et al., 2020; Elad et al., 2022; Zhang et al., 2022). Random mutagenesis of the y*qjF* promotor resulted in an enhanced signal with effector molecules, and mutations on the YhaJ-coding region increased the signal, too (Shemer et al., 2020; Elad et al., 2022). A degradation tag to the components showed highly decreased noise, although this modification also reduced the fluorescent signal (Zhang et al., 2022).

Recently, we reported the crystal structures of the effector binding domain (EBD) of YhaJ and its complex with an effector (Kim et al., 2023; Kim et al., 2024). The structures revealed detailed interactions between YhaJ and the effector, indicating a novel effector binding mechanism involving a loop switch at the entrance of an effector binding pocket. The effector binding mechanism-based mutation exhibited an increased effector binding affinity and response ratio (Kim et al., 2023). Here, we determined the crystal structure of the YhaJ DNA binding domain (DBD) together with the linker helix mediating a dimeric structure. A modeling study based on the BenM-DBD:DNA complex structure provided insights into DNA recognition by YhaJ-DBD. Mutations aiming at increasing the charge interaction between the negative-charged phosphates and DBD yielded a mutation that increased the DNA binding affinity. Unexpectedly, the increase of DNA binding affinity resulted in a signal leakage decrease instead of increasing the signal itself. The signal leakage repression resulted in a significant increase in the response ratio for biosensor performance. The observation explains a novel regulation mechanism of LTTRs, providing insights for the development of high-performance biosensors by using the signal leakage minimization strategy.

## 2 Materials and methods

### 2.1 Expression and purification

YhaJ-DBD (residues 1-93; Uniprot P67661) was amplified from the lysate of *E. coli* strain K-12 and cloned into the pET-21b vector. The recombinant protein was overexpressed using *E. coli* BL21(DE3). Cells were cultured at 37°C until OD600 reached 0.6, and the protein expression was induced by 0.1 mM isopropyl β-D-1-thiogalactopyranoside. After incubation at 18°C overnight, cells were harvested and lysed with sonication. The solution containing soluble proteins was separated by centrifugation for an hour at 4°C, and loaded into the nickel affinity chromatography column. The column was washed with the wash buffer (50 mM Tris-Cl pH 7.5 and 1.0 M NaCl) and the buffer containing imidazole (50 mM Tris-Cl pH 7.5, 0.5 M NaCl, 30 mM imidazole, and 10 mM 2-mercaptoethanol) in turn. The His-tagged protein was eluted by a buffer with a high concentration of imidazole (50 mM Tris-Cl pH 7.5, 0.2 M NaCl, 0.5 M imidazole, and 5 mM 2-mercaptoethanol). The protein fractions were pooled, and injected into the Sephacryl S-100 column (Cytiva) equilibrated with a final buffer (20 mM Tris-Cl pH 7.5, 150 mM NaCl, and 10 mM 2-mercaptoethanol). All purification steps were conducted at 4°C. The protein was concentrated to 30 mg/mL and frozen at −70°C.

### 2.2 Crystallization and structure determination

Crystallization trials were carried out at 18°C with the sitting-drop vapor-diffusion method. The best crystals for diffraction were obtained from a 0.3 μL reservoir buffer (0.1 M Tris-Cl pH 8.5 and 2.0 M ammonium phosphate monobasic) and an equal volume of the 30 mg/mL protein solution. Crystals appeared within a day. Diffraction data were collected on beamline 7A at Pohang Accelerator Laboratory under 100 K cryo-stream. The crystal was cryo-protected by soaking it in the mother liquor containing 20% glycerol. Diffraction images were processed by the software HKL-2000 (Otwinowski and Minor, 1997), generating the merged file. The merged data were analyzed by Xtriage in Phenix (Zwart et al., 2008), showing two macromolecules in the asymmetric unit. The structure was solved by the molecular replacement using the program Phaser in Phenix (Zwart et al., 2008). A model of YhaJ-DBD from the AlphaFold Protein Structure Database (residues 6-93; AF-P67661-F1) (Varadi et al., 2022) was used as the initial search model. The structure solution from the molecular replacement was refined with iterative rounds of manual building using the program Coot (Emsley and Cowtan, 2004) and refinement using the program phenix.refine. Positioning phosphate and chloride ions was conducted by the program Phenix (Zwart et al., 2008).

### 2.3 Molecular modeling

The initial model for molecular dynamics (MD) was prepared by superposing the crystal structure of YhaJ-DBD dimer with the crystal structure of BenM-DBD complexed with its target DNA (PDB ID 4IHT) using the program Coot (Emsley and Cowtan, 2004). The DNA sequence for the target DNA model was manually mutated by the Coot (Emsley and Cowtan, 2004). For MD simulation, the QwikMD extension in Visual Molecular Dynamics (Ribeiro et al., 2016) was utilized. The NAMD v2.14 software (Phillips et al., 2005) with the CHARMM36 force field (Croitoru et al., 2021) was applied to conduct the simulation. The YhaJ-DNA model was solvated with TIP3P cubic water box under an explicit solvent option and 0.15 mol/L NaCl condition. The simulation was calculated to 10 ns at 300K with the protocol of 2,000 minimization steps, 144,000 annealing steps, and 500,000 equilibration steps. The resulting model was analyzed by QwikMD analysis tools (Ribeiro et al., 2016) and the Coot (Emsley and Cowtan, 2004).

### 2.4 Protein-DNA affinity measurement

The C55 promoter gene (Elad et al., 2022) synthesized by Bioneer Inc., was amplified by PCR using biotinylated primers, resulting in double-strand oligonucleotides of 250 bp length. The PCR product was gel-purified and diluted with TBS (20 mM Tris-Cl pH 7.5 and 150 mM NaCl) to a final concentration of 5 nM, loaded to 96-well Pierce™ Streptavidin Coated Plates (Thermo Scientific). After removing the unbound DNA, a solution containing 15 and 45 μM of c-Myc-tagged YhaJ-DBD proteins was transferred to each well and incubated for 2 h at room temperature. Then, anti-c-Myc antibody (clone 9E10; Santa Cruz Biotechnology) and anti-Mouse IgG(H + L)-HRP (SA001-500; GenDEPOT) were treated in turn. The wells were washed more than 3 times using TBS buffer after each step. The bound antibody was detected using a TMB solution. The absorbance signal was measured using EMax Microplate Reader (Molecular Devices) at 450 nm wavelength.

### 2.5 GFP-based cell assay

The assay was conducted as previously reported with some modifications (Kim et al., 2023). The oligonucleotides of *yhaJ* open reading frame and *yqjF* promoter linked to GFPmut2 gene were synthesized (Bioneer) and cloned into the pET-Duet-1 vector (Novagen), replacing genetic elements of the vector required for protein overexpression. Mutations were introduced by QuikChange Site-directed Mutagenesis protocol (Stratagene). *E. coli* TOP10 cells (Invitrogen) chemically transformed with plasmids were used for the experiments. Colonies were cultured overnight in LB media supplemented with 50 μg/mL ampicillin. Cultures were diluted and grown in LB media at 37°C. After some hours, a final concentration of 1% (v/v) DMSO or MHQ (Tokyo Chemical Industry) dissolved in DMSO was added to cultures. The relative fluorescence unit (RFU) and OD_600_ values were measured by transferring 200 μL of culture to 96-well black plates and transparent plates (SPL Life Sciences), respectively. The fluorescence from GFP was measured using Perkin-Elmer Victor X2 device at excitation/emission wavelengths of 485/535 nm, respectively.

## 3 Results and discussion

### 3.1 Structure of YhaJ-DBD

**FIGURE 1:**
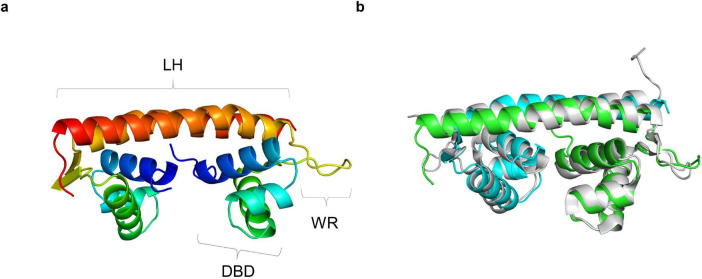
Crystal structure of YhaJ-DBD. **(a)** The dimeric structure of YhaJ-DBD was presented. Each monomer was shown in rainbow colors (blue for the N-terminus and red for the C-terminus). The DBD (DNA-binding domain), WR (winged region), and LH (linker helix) were labeled with brackets. **(b)** Superposition of the crystal structure of YhaJ-DBD dimer (green for chain **a**; cyan for chain **b**) with that of BenM-DBD dimer (gray; PDB ID 4IHS).

The YhaJ DNA binding domain linked to a linker region (named YhaJ-DBD, residues 1-93) with a C-terminus hexahistidine tag was purified using affinity chromatography and size exclusion chromatography. The purified recombinant protein was a dimer in solution as determined by an analytical size exclusion chromatography (data not shown). The crystal structure of YhaJ-DBD was determined at 1.76 Å resolution and refined to R-work and R-free values of 17.3 and 21.8%, respectively (Table 1). The asymmetric unit contained a dimer of YhaJ-DBD’s. The YhaJ-DBD structure adopts a winged-helix-turn-helix fold typically seen in LTTR family proteins (Figure 1A). Three α-helices (α1 [Leu9-Arg21], α2 [Phe24-Gly32], and α3 [Pro35-Asp50]) constitute the DNA binding region, followed by a winged region (WR) (Val51-Thr64) and a long linker helix (LH) (α4 [Asn65-Arg93]). Hydrophobic residues in the α1 - α3 helices (α1 [Ala11, Leu12, Val14, Met15, and Ile18], α2 [Phe24, Ala27, and Leu31], and α3 [Met42, Leu45, and Leu49]) form a strong hydrophobic core of the DBD. Residues in the LH of one monomer interact with those of the other monomer mainly by hydrophobic interactions. In the dimer, two charged residues Arg73 and Asp87 in one LH interact with Asp87 and Arg73 of the other LH, respectively, stabilizing an anti-parallel orientation of LH’s.

**TABLE 1:** Data collection and refinement.

Wavelength (Å)	0.9793
Resolution range (Å)	33.16 – 1.76 (1.82 – 1.76)
Space group	H3
**Unit cell**	
a, b, c (Å)	117.16, 117.16, 35.09
α, β, γ (°)	90.00, 90.00, 120.00
Total number of reflections	65383
Unique reflections	17579
Redundancy	3.7
Completeness (%)	98.86 (95.05)
I/σI	13.59 (4.25)
R_merge_	0.062 (0.223)
CC_1/2_	0.997 (0.873)
Reflections used in refinement	17576
Reflections used for R-free	1759
R-work/R-free	17.3%/21.8%
**Number of total atoms**	
Protein/ligand/water	1472/16/121
Protein residues	187
RMS (bonds) (Å)	0.006
RMS (angles) (°)	0.87
**Ramachandran plot (%)**	
Favored/allowed/outliers	98.9/1.1/0.0
**Average B-factor**	
Protein/ligand/water	24.07/42.62/31.23

### 3.2 Modeling of DNA-binding interaction

**FIGURE 2:**
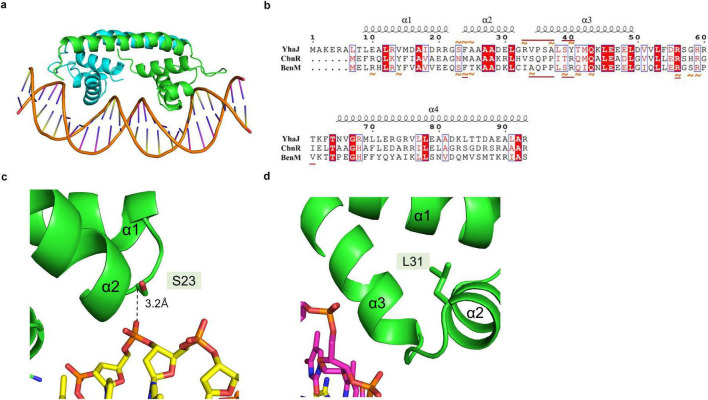
Modeled structure of the YhaJ-DBD and a YhaJ binding motif complex. **(a)** The overall structure of the modeled complex of YhaJ-DBD:DNA was presented in a ribbon diagram. Each YhaJ-DBD monomer was colored green and cyan, and the DNA backbone was displayed using an orange color. The base moieties of the oligonucleotide were represented in a stick shape. **(b)** Interactions between LTTR amino acids and the target DNA were presented on the aligned sequences of YhaJ, CbnR, and BenM. The red amino acid codes in blue squares represent homologous sequences, and white amino acid codes shaded in red mean identical sequences across the three LTTRs. The orange tildes designate the interaction with a backbone, while the straight underlines in dark red show the interaction with a base moiety. The interaction in BenM-benA was based on Alanazi et al. (2013). The interaction in YhaJ-yqjF was defined by atom distances within 4.5 Å while ignoring that of the winged region with high uncertainty. The secondary structure elements were displayed according to those of YhaJ. The image was generated using the program Espript 3 (Robert and Gouet, 2014). **(c,d)** Close-up views of the regions for panel **(c)** Ser23 and **(d)** Leu31 residue. The side chains and oligonucleotides were presented in a stick diagram. The distance between Ser23 Cα and a phosphate group on the DNA backbone was shown using a dashed line.

The structural model for the YhaJ-DBD dimer bound to a double-strand DNA was built by superposing the YhaJ-DBD with a previously reported structure of the LTTR:DNA complex. To date, LTTR structures of BenM and CbnR were determined as complex with their target DNA molecules (Alanazi et al., 2013; Koentjoro et al., 2018). When the YhaJ-DBD was superposed with the corresponding region of BenM (PDB code: 4IHS) (Figure 1B), the average Cα RMSD with YhaJ-DBD was 0.397 Å which was lower than that of CbnR (0.998 Å for PDB code: 5XXP). Thus, we chose the BenM:DNA complex for the modeling of the YhaJ:DNA complex. The prospective YhaJ binding region of the C55 promoter (Elad et al., 2022) was utilized as an oligonucleotide for the YhaJ:DNA complex. The molecular dynamics (MD) simulation was conducted using the initial YhaJ:DNA complex for 10 ns. There were little changes in RMSD values in the structure throughout the trajectory, which indicated stable interactions between YhaJ and DNA in the complex model (Figure 2A).

In the YhaJ-DBD:DNA complex model, YhaJ-DBD interacts with the major groove of the target DNA. Due to the ambiguity of a palindromic sequence in the binding motif of YhaJ promotor (Palevsky et al., 2016), we mainly focused on the DNA backbone phosphate interactions with the YhaJ-DBD residues. Although the structures of YhaJ, CbnR, and BenM are well aligned with Cα-carbon superpositions, amino acid residue types and projection direction of their side chains are quite variable, resulting in differences in the DBD:DNA interactions in different LTTRs (Figure 2B). These difference would allow each LTTR’s unique DNA recognition mode and sequence specificity. Positive residues (Arg33, Lys44, Arg56, and Arg60) of YhaJ-DBD interact with negative-charged phosphate groups in nucleotides. There are non-charged residues whose side chains are toward the DNA phosphate groups. For example, the Cα-carbon of Ser23 is 3.2Å away from the DNA phosphate atom, indicating potential interactions (Figure 2C). The G2 variant, which showed the YhaJ effector response enhancement, included one mutation (Leu31) in the DBD region (Elad et al., 2022). Interestingly, the position of Leu31 is in the hydrophobic core of the helix-turn-helix motif (Figure 2D), excluding the possibility of direct DNA interactions. The mutation at Leu31 likely affects the conformation of helix α3 which has DNA interactions.

### 3.3 Mutant screening for high-affinity DNA binding

**FIGURE 3:**
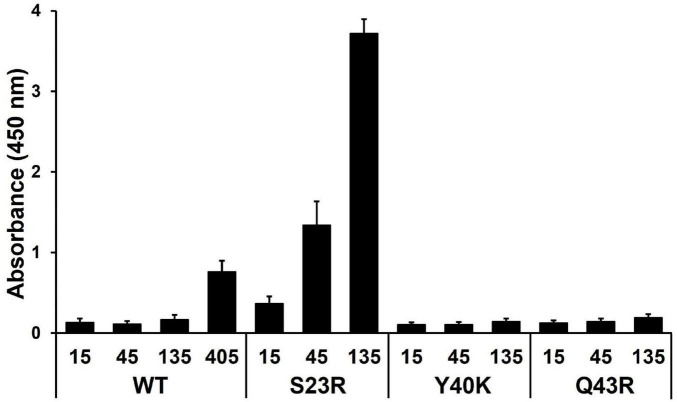
Measurement of YhaJ-DBD binding affinity to the promoter gene. The binding affinity of YhaJ-DBD WT, S23R, Y40K, and Q43R to the C55 promoter gene was analyzed. The *X*-axis shows YhaJ protein status and their concentrations. The *Y*-axis represents the absorbance signal induced by the HRP-conjugated antibody bound to YhaJ-DBD proteins. Each measurement was in triplicate, and the standard deviation is a bar on the block representing the average value. The WT measurement included a high concentration of 405 μM because the WT did not show a signal at lower concentrations.

We hypothesized that strengthening YhaJ-DBD:DNA interactions by mutating the interacting residues may enhance the detection of explosives. We inspected the interaction interface of the YhaJ-DBD:DNA complex model and found several non-charged residues of YhaJ are close to the DNA phosphate backbone (Figure 2B). Mutations conferring a positive charge on those residues may have the potential for increased complex-formation affinity by charge interactions with the negatively charged phosphate. We mutated several of those to arginine or lysine and measured the binding affinity between the mutated YhaJ-DBD and DNA (Figure 3). In the affinity measurement, the S23R mutant YhaJ-DBD protein showed significant binding to the biotin-labeled YhaJ promotor. Other mutants showed either very weak (Q43R) or no binding affinity (Y40K) (Figure 3). The wild-type YhaJ-DBD also showed weak interaction with the promotor. The wild-type showed interaction at the concentration of 405 μM, whereas the S23R interacted strongly at the concentration of 15 μM and above.

The binding affinities of the S23R and Q43R mutants and the wild type were dose-dependent, indicating the validity of the affinity measurement assay. In the affinity assay, we used the C55 promoter which was obtained by optimizing mutations of the YhaJ promotor (Elad et al., 2022). The YhaJ-binding region of C55 contains base mutations in the YhaJ binding sequence region (Elad et al., 2022) which disrupt the palindromic sequence in the native sequence (Palevsky et al., 2016). The symmetry breakage in the palindromic sequence may contribute to the weak binding of the wild-type YhaJ-DBD by decreasing interactions with DNA bases. The bacterial promotor efficiency can be affected by −10, −35, UP elements, and various transcription factor bindings (Estrem et al., 1999; Leuze et al., 2012). The enhanced effector response of C55 is likely due to the optimization of regions other rather than the direct binding of YhaJ-DBD. The stronger phosphate backbone interactions in the S23R and Q43R mutants likely overcome the weakened base interactions of C55. However, the definite explanation of the affinity increase mechanism needs a high-resolution structure of the YhaJ-DBD:DNA complex.

### 3.4 *In vivo* activity

**FIGURE 4:**
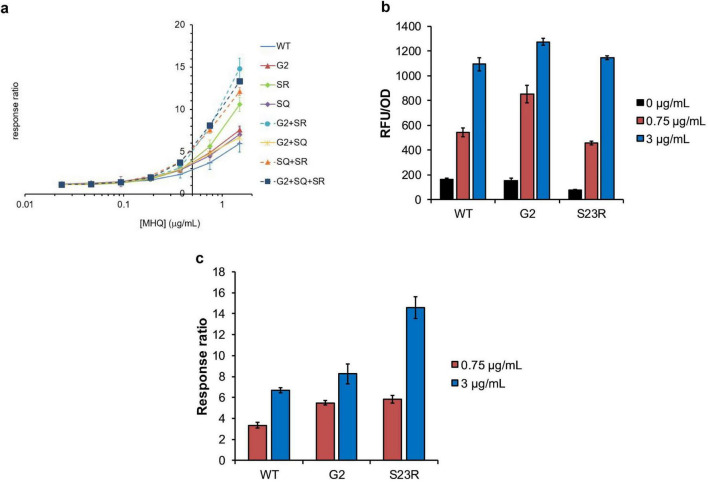
*In vivo* activity of YhaJ mutants. **(a)** The signal (*Y*-axis) induced by MHQ of various concentrations (μg/mL) in a logarithmic scale (*X*-axis) was displayed as the response ratio for each YhaJ mutant. In the figure, SR and SQ represent the S23R mutant and the S267Q mutant, respectively. The dots and error bars represent the mean ± SD of the response ratio for three experiments. **(b)** The relative fluorescence unit (RFU/OD) values induced by different concentrations of MHQ in the mutant screening experiment are presented for YhaJ-DBD WT, G2, and S23R. The blocks and error bars represent the mean ± SD of the RFU/OD for three experiments. **(a,b)** are independent experiments. **(c)** The values in panel **(b)** were shown as the response ratio for YhaJ-DBD WT, G2, and S23R. The blocks and error bars represent the mean ± SD of the response ratio for three experiments.

We conducted a green fluorescent protein (GFP)-based cell assay to evaluate the effect of mutations on effecter responsiveness. One of the YhaJ effectors methylhydroquinone (MHQ), a bacterial metabolite of DNT, was used for the assay. MHQ elicits a strong response when treated to the YhaJ-containing reporter systems (Henshke et al., 2021). Consistent with the binding affinity assay, the S23R mutation significantly enhanced the response ratio (fluorescence in the presence of MHQ divided by that in its absence) (Figure 4A). At the MHQ concentration of 1.5 μg/mL, the response ratio of the S23R mutant was 10.6 which was about 1.8 fold greater than that of the wild type (6.0). The response ratio of the S23R mutant was significantly greater than that of the previously found mutations including the G2 (Elad et al., 2022) and S267Q (Kim et al., 2023) mutations. When the S23R mutant combined with the G2 or S267Q mutations, the response ratio significantly increased. The G2 + S267Q + S23R or G2 + S23R combinations exhibited the highest response ratio. Thus, the DNA binding mechanism-based S23R mutation synergizes with previously found mutations. The S267Q mutant increases the ligand binding affinity by destabilization of the entrance loop for the ligand binding pocket (Kim et al., 2023). The modeled structure of the YhaJ-DBD:DNA complex indicates that the S23R mutant increases the interaction by introducing a charge interaction (Figure 2C). Although the structural mechanism of G2 is not completely understood, the effects of S267Q and S23R mutants indicate that the activation of the YhaJ promotor can be modulated by structure-based engineering of both the ligand-binding and DNA-binding domains.

Interestingly, the response ratio enhancement of the S23R mutation mainly resulted from the decreased signal for the effector-absent state (Figure 4B). In the absence of the effector (0 μg/mL bars in Figure 4B), the transcription signal of the S23R mutant (70) is about half of the wild type (164). The signals are similar (1145 vs 1094) in the presence of a 3 μg/mL effector. The signal decrease in the absence of an effector resulted in about two times the response ratio for the S23R mutant compared to the wild type in the presence of a 3 μg/mL effector (Figure 4C). In the case of G2, the signal is almost unchanged in the absence of effector compared to the wild type, and it increases in the presence of a 3 μg/mL effector, resulting in a little increase of the response ratio (Figure 4C). Thus, the performance of the YhaJ-based biosensor, which is the response ratio, can be enhanced either by increasing the director effector response as well as by decreasing the signal leakage at the absence of an effector.

**FIGURE 5:**
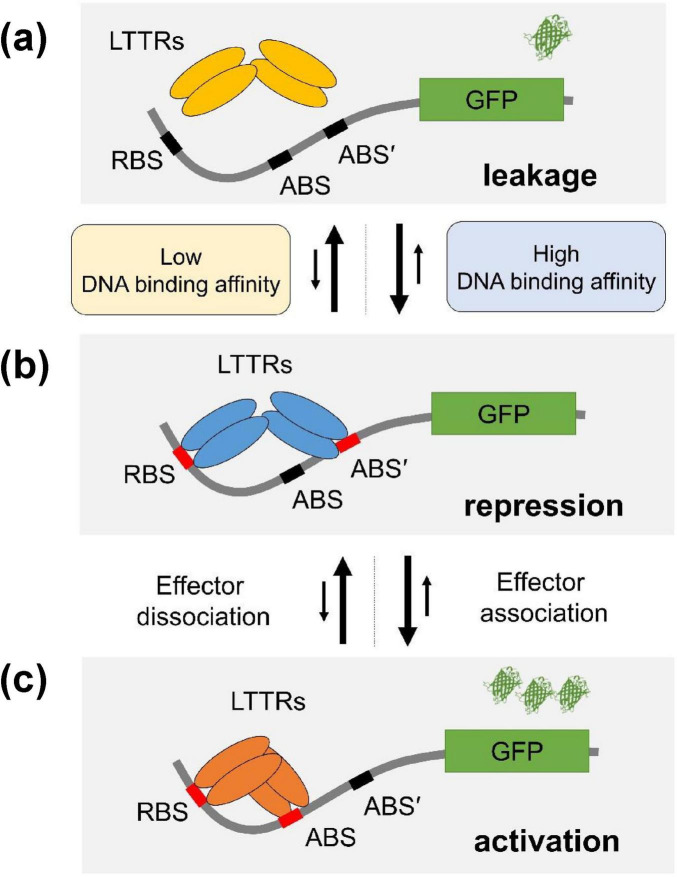
Mechanism of improvement of response ratio. The mechanism of the YhaJ mutant improving sensor performance was illustrated. YhaJ homotetramer (represented as LTTRs in the figure) was depicted as ovals. RBS, ABS, and ABS’ binding sites for YhaJ DBD represent the recognition binding site, the activation binding site, and the secondary activation binding site, respectively. LTTRs shift their DNA binding from ABS’ to ABS in the presence of an effector. **(a)** The signal leakage state, **(b)** the repression state, and **(c)** the activation state. The stronger binding of the YhaJ mutant to DNA stabilizes the repression state. The number of β-barrel-shaped green fluorescent protein (GPF) indicates different expression levels of reporter protein molecules. The proposed YhaJ binding motif in the YhaJ-binding promotor *yqjF* is TCAAATTTTTTGAAGA which is similar to the typical LTTR binding sequence motif T-N11-A (Palevsky et al., 2016). However, the definite assignment of RBS, ABS, and ABS’ sequences in the YhaJ promotor has not been reported yet.

LTTR transcription factors function as a tetramer that comprises two DNA-binding units (Figure 5). One of the DNA-binding domains binds to the regulatory binding site (RBS) with high affinity, and the other binds to the activation binding site (ABS) with low affinity (Maddocks and Oyston, 2008; Baugh et al., 2023). The promotors for LTTRs have basal-level transcription activities even when there is no transcription factor interaction (Figure 5A). In the LTTR promotors, there are two ABS sites (ABS and ABS’) where ABS’ is occupied by the LTTR DBD in the absence of an effector (Figure 5B), and ABS is occupied in the presence of an effector (Figure 5C; Baugh et al., 2023; Alanazi et al., 2013). To repress the basal level transcription, LTTRs bind to both RBS and ABS’ (Figure 5B). When effectors bind to the effector binding domain of LTTRs, the tetrameric orientation is changed, and the tetramer binds to RBS and ABS (Figure 5C) instead of RBS and ABS’. The S23R mutation seems to stabilize the repression state (Figure 5B) due to the increased DNA binding affinity of the mutant. In Figure 3, we showed that the DNA binding affinity of the S23R mutant was significantly increased compared to that of the wild type. The strong DNA binding likely has correlation with the decrease of the basal-level activity shown in Figure 4B. Stabilization of the repression state shifts the equilibrium between the leakage and repression states toward the repression state, resulting in a decreased signal leakage.

## 4 Conclusion

The structure of the DNA-binding domain (DBD) of YhaJ and mutagenesis screening found that the S23R mutation of the YhaJ-DBD increased the DNA binding affinity. The S23R mutant exhibited a significantly enhanced response ratio compared to the wild-type protein. Interestingly, the mutant had a similar effector response intensity to the wild type. However, the mutant decreased the signal leakage to about half of the wild type, resulting in a significant enhancement of the response ratio regarded as a representative factor for the biosensor performance. The biosensor performance enhancement by the signal leakage repression combines with the previous signal-enhancing mutations. Thus, the promotor binding affinity of LTTRs fine-tunes the bacterial transcription repression and activation, and both the signal enhancement and the signal leakage repression are means for the biosensor performance enhancement of LTTRs.

## Data Availability

The datasets presented in this study can be found in online repositories. The names of the repository/repositories and accession number(s) can be found in this article/supplementary material.
